# Expression Analysis of Key Auxin Biosynthesis, Transport, and Metabolism Genes of *Betula pendula* with Special Emphasis on Figured Wood Formation in Karelian Birch

**DOI:** 10.3390/plants9111406

**Published:** 2020-10-22

**Authors:** Tatiana V. Tarelkina, Ludmila L. Novitskaya, Natalia A. Galibina, Yulia L. Moshchenskaya, Kseniya M. Nikerova, Nadezhda N. Nikolaeva, Irina N. Sofronova, Diana S. Ivanova, Ludmila I. Semenova

**Affiliations:** Karelian Research Centre of the Russian Academy of Sciences, Forest Research Institute, 11 Pushkinskaya st., 185910 Petrozavodsk, Russia; ludnovits@rambler.ru (L.L.N.); galibina@krc.karelia.ru (N.A.G.); tselishcheva.yulia@mail.ru (Y.L.M.); knikerova@yandex.ru (K.M.N.); kar-birch@mail.ru (N.N.N.); irinasof@krc.karelia.ru (I.N.S.); dszapevalova@mail.ru (D.S.I.); mi7enova@gmail.com (L.I.S.)

**Keywords:** auxin, *Yucca*, *PIN*, *GH3*, figured wood formation, xylem vessels

## Abstract

Auxin status in woody plants is believed to be a critical factor for the quantity and quality of the wood formed. It has been previously demonstrated that figured wood formation in Karelian birch (*Betula pendula* Roth var. *carelica* (Merckl.) Hämet-Ahti) is associated with a reduced auxin level and elevated sugar content in the differentiating xylem, but the molecular mechanisms of the abnormal xylogenesis remained largely unclear. We have identified genes involved in auxin biosynthesis (*Yucca*), polar auxin transport (*PIN*) and the conjugation of auxin with amino acids (*GH3*) and UDP-glucose (*UGT84B1*) in the *B. pendula* genome, and analysed their expression in trunk tissues of trees differing in wood structure. Almost all the investigated genes were overexpressed in Karelian birch trunks. Although *Yucca* genes were overexpressed, trunk tissues in areas developing figured grain had traits of an auxin-deficient phenotype. Overexpression of *GH3*s and *UGT84B1* appears to have a greater effect on figured wood formation. Analysis of promoters of the differentially expressed genes revealed a large number of binding sites with various transcription factors associated with auxin and sugar signalling. These data agree with the hypothesis that anomalous figured wood formation in Karelian birch may be associated with the sugar induction of auxin conjugation.

## 1. Introduction

The hormone auxin is involved in regulating virtually all morphogenetic processes in plant organisms, and its role in conducting tissues differentiation in tree trunks is well known [1,2,3,4,5,6,7,8]. Auxin contribution to the regulation of morphogenetic processes largely depends on its concentration and distribution in plant organs and tissues. The level of free (physiologically active) auxin in cells and tissues is the result of an intricate crosstalk of processes for its transport and metabolism, which, in turn, includes hormone biosynthesis, degradation and conjugation [9,10,11]. 

The principal gene families involved in IAA (the main natural auxin form) homeostasis in tissues have been identified. Much of IAA in plants is synthesised from tryptophan with the involvement of Yucca flavin-dependent monooxygenases [11]. *Yucca* family genes have been detected in many plants, including woody plants—*Populus trichocarpa* [12], *Vitis vinifera* [13], *Prunus persica* [14], *Eucalyptus globulus* [15] and *Malus domestica* [16,17]. It has been shown that de novo biosynthesis of auxin in various organs and tissues is an important player in generating the local gradients of the hormone [18,19,20].

Polar auxin transport is responsible for the elongate prosenchymatous shape of conducting tissue cells and is required for the differentiation of the most specialised conducting elements of the xylem vessels [21,22,23,24,25,26,27,28]. Proteins involved in polar auxin transport are encoded by *PIN* family genes [29]. Two groups of PIN proteins are distinguished based on their structural organisation and localisation in the cell. So-called “long” PINs contain a long hydrophilic domain localised between two transmembrane domains; they are localised on the plasmalemma and participate in auxin efflux from the cell. “Short” PINs have a reduced hydrophilic domain and are localised in the endoplasmic reticulum (ER) membrane [30,31]. There is some evidence that the PIN6 protein, which has the hydrophilic loop of intermediate length in comparison with “long” and “short” PINs, is localised on the plasmalemma as well as in the ER membrane [32,33]. *PIN* family genes have been identified in a number of woody plants—*Picea abies* [34], *Populus sp.* [32,35], *E. globulus* [15], *M. domestica* [16], *V. vinifera* [36], *Pinus radiata* [37] and *Pyrus bretschneideri* [38]. 

Genes of the *GH3* family encode the enzymes contributing to the conjugation of auxin and some other plant hormones with amino acids to produce amide conjugates [9,39]. *GH3* genes have been detected in many woody plants, such as *P. trichocarpa* [40], *M. domestica* [41], *Castanea sativa* [42], *Betula platyphylla* [43], *P. abies* [44], and *Larix leptolepis* × *Larix olgensis* [45]. Some plants were also found to have genes encoding UDP-glycosyl transferases (UGT), which participate in IAA conjugation with UDP-glucose [46,47,48]. Auxin conjugates are physiologically inactive, and have no role in polar transport of the hormone [9,39,49]. It is believed that different forms of conjugates perform different functions. Ester conjugates as well as some amide conjugates (IAA–Ala, IAA–Leu, IAA–Phe) are the forms for non-polar hormone transport and storage, which can be cleaved to release free IAA [9,49,50]. In contrast, IAA–Asp and IAA–Glu are suggested to be precursors for the degradation pathway [39,49]. Some data indicate that IAA–Trp is an auxin antagonist, affecting its signalling [51].

Although the role of auxin in conducting tissues formation has been known for quite a while and the key genes involved in its homeostasis have been identified in some woody plants, papers investigating the molecular–genetic aspects of auxin biosynthesis, transport and inactivation in woody plant trunk tissues are scarce. Furthermore, the activity of the genes in woody plants has been chiefly studied in trees younger than 5 years [12,16,17,41,42,43,52,53]. To the best of our knowledge, there have been no previous integrated studies of the activity of the genes involved in auxin homeostasis in trunk tissues of naturally growing older trees.

Silver birch (*Betula pendula* Roth) is a fast-growing woody species and an important source of timber in Northern Europe [54,55]. A naturally occurring silver birch variety—Karelian or curly birch (*Betula pendula* Roth var. *carelica* (Merckl.) Hämet-Ahti)—forms highly valuable figured wood [56,57,58]. Figured wood of Karelian birch resembles “wooden marble” in appearance and is one of the most expensive timbers in Europe. The ability to develop figured wood is hereditary, but even the progeny obtained through controlled pollination of plus trees would always contain straight-grained individuals—so-called non-figured Karelian birch trees [59,60,61,62].

Wide variation of wood structure makes birch an interesting object for studying mechanisms regulating xylogenesis. The figured wood of Karelian birch is different from the typical straight-grained wood of silver birch not only in appearance, but also in the ratio of cell types. While the dominant elements in common silver birch wood are those with the water conducting and mechanical functions—fibrous tracheids and vessels—the figured wood of Karelian birch mainly consists of radial and axial parenchyma [58,59,63]. Research into biochemical aspects revealed that figured wood in Karelian birch forms in the context of a reduced level of free (physiologically active) auxin and a high concentration of sucrose in trunk tissues, which is degraded involving cell wall invertase, while birch trees forming wood typical of the species have a higher auxin level, and sucrose in their trunk tissues is primarily metabolised by sucrose synthase [58,63,64,65,66,67,68]. The molecular groundwork of interactions between auxin and sugars in the process of anomalous wood formation in Karelian birch, however, has so far been studied very little [69]. Meanwhile, such studies are of high theoretical as well as practical interest, since variants of abnormal xylogenesis occur in a number of other woody plants, too. E.g., the formation of spiral grain in *Pinus sylvestris* wood is connected with auxin distribution and transport in trunk tissues [2,70,71,72,73,74] and also is associated with a reduced sucrose synthase activity and an elevated cell wall invertase activity in the differentiating xylem [75].

A hypothesis has recently been proposed that figured wood formation in Karelian birch may have to do with inactivation of free auxin as a result of auxin conjugation which, via a chain of biochemical reactions, may be associated with hexoses produced in the apoplast through sucrose cleavage by cell wall invertase [69]. We know, however, that sugars can modulate auxin metabolism and transport both by acting as substrates in conjugation reactions and by regulating gene expression [39,76,77,78]. To elucidate how auxin homeostasis can be affected during figured wood formation, we identified the genes encoding the main auxin biosynthesis, transport and conjugation enzymes and studied their expression patterns in various xylogenesis scenarios in silver birch. The objects were common silver birch, non-figured and figured Karelian birch trees.

## 2. Results

### 2.1. The Anatomy of Conducting Tissues in Sample Tree Trunks

**Figure 1:**
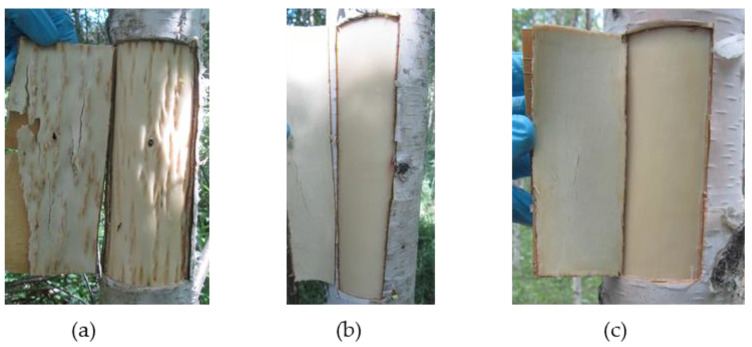
Appearance of trunk tissues (wood and bark) in sampled trees: (**a**) figured *B. pendula* var. *carelica* (Bc FT) tree; (**b**) non-figured *B. pendula* var. *carelica* (Bc NF) tree; (**c**) *B. pendula* var. *pendula* (Bp) tree.

Visual observation revealed no signs of anomalous structure in the trunk tissues of common silver birch trees (*B. pendula* var. *pendula*, hereafter Bp trees) and Karelian birch trees with straight-grained non-figured wood (non-figured *B. pendula* var. *carelica* trees, hereafter Bc NF trees), whereas Karelian birch trees with richly figured wood (figured *B. pendula* var. *carelica* trees, hereafter Bc FT trees) demonstrated pronounced anomalies in wood and bark structure (Figure 1). 

**Figure 2:**
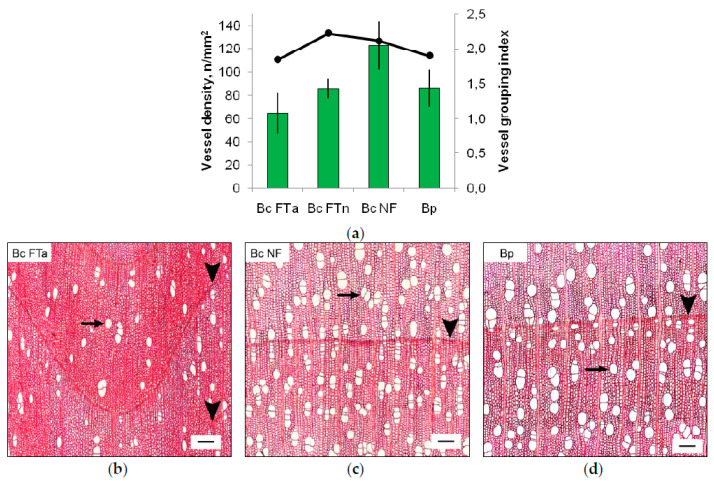
Parameters and appearance of xylem tissue in sampled birch trees: (**a**) vessel density and vessel grouping index (indicated by the line chart). Vessel grouping index is defined as the ratio of the total number of vessels to the total number of vessel groupings (incl. solitary and grouped vessels). FTa and FTn indicate figured and non-figured parts from figured *B. pendula* var. *carelica* tree trunks; (b, c, d) transverse sections of the xylem: (**b**) figured *B. pendula* var. *carelica* (Bc FT) tree; (**c**) non-figured *B. pendula* var. *carelica* (Bc NF) tree; (**d**) *B. pendula* var. *pendula* (Bp) tree. Black arrowhead points to the annual increment outline, black arrow indicates vessels. Scale bar = 100 µm.

Abnormal wood in trunk areas with figured grain (Bc FTa parts) has been forming over at least the last three growing seasons. The main traits of an abnormal xylem structure were a tortuous annual increment outline and a reduced density of vessels (Figure 2). The number of vessels per unit area was the greatest in Karelian birch xylem with a normal structure (in Bc NF trees and in trunk areas with non-figured wood (Bc FTn parts)). The lowest values of this parameter were found in Bc FTa parts. The greater number of vessels in Karelian birch xylem with a normal structure ensued from a larger proportion of vessels in groups (radial multiples and clusters). We saw no large parenchyma aggregations or pronounced curling of structural elements in the xylem in Bc FTa parts. 

**Figure 3:**
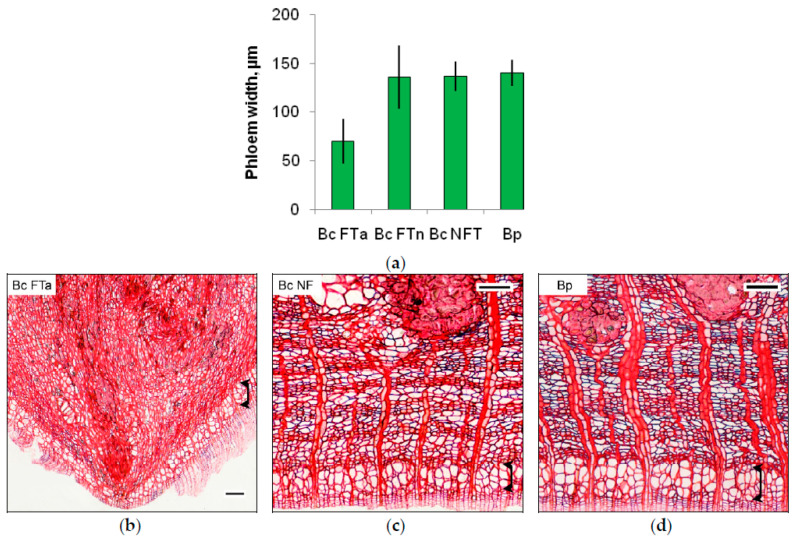
Conducting phloem width (**a**) and appearance of secondary phloem tissue in sampled birch trees (transverse section): (**b**) figured *B. pendula* var. *carelica* (Bc FT) tree; (**c**) non-figured *B. pendula* var. *carelica* (Bc NF) tree; (**d**) *B. pendula* var. *pendula* (Bp) tree. FTa and FTn indicate figured and non-figured parts from figured *B. pendula* var. *carelica* tree trunks. Conducting phloem is indicated by the rounded double arrow. Scale bar = 100 µm.

Bark tissues in Bc FTn parts, as well as in Bc NF trees and Bp trees, had a structure typical of the species, with no signs of anomalies. The bark in Bc FTa parts was thicker than in non-figured parts of the same trunk. At the same time, conducting phloem width in anomaly areas was half its width in the bark formed in the variants with straight-grained wood. The reduced conducting phloem thickness in Bc FTa parts was a consequence of a smaller number of sieve elements (2–3 cells in annual phloem increment) compared to other variants (5–6 cells in annual phloem increment) (Figure 3). 

### 2.2. Yucca and GH3 Identification in the Silver Birch Genome

#### 2.2.1. Yucca Family Genes

**Figure 4:**
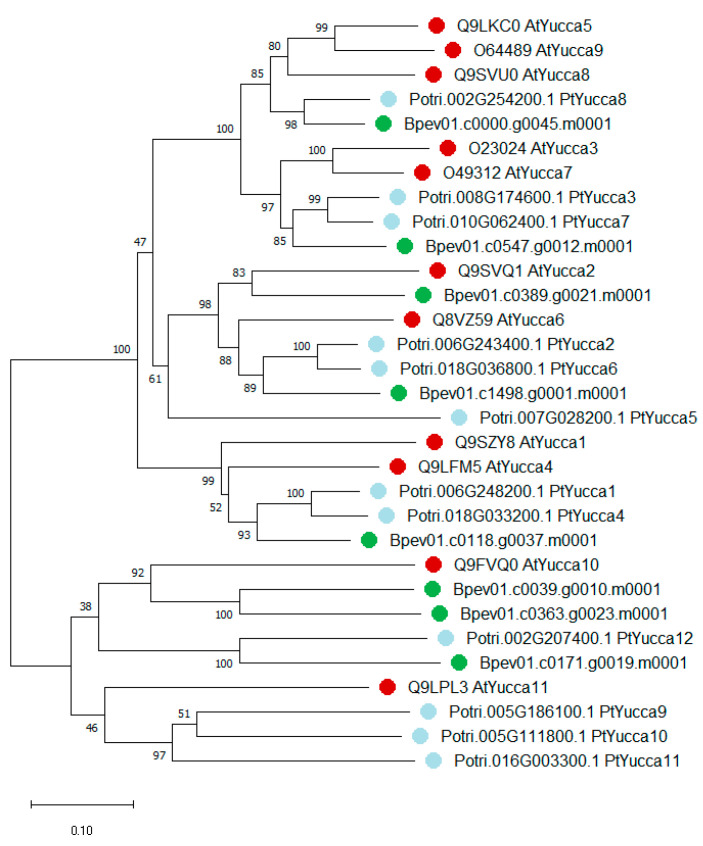
Phylogenetic relationships of Yucca proteins of *B. pendula* (green dots), *A. thaliana* (red dots) and *P. trichocarpa* (blue dots). The tree is drawn to scale, with branch lengths in the same units as those of the evolutionary distances used to infer the phylogenetic tree. Bootstrap values (1000 replicates) are shown next to the branches. The access codes of the *A. thaliana* and *P. trichocarpa* proteins in the UniProt/SwissProt and Phytozome databases are indicated next to the corresponding proteins.

**Figure 5:**
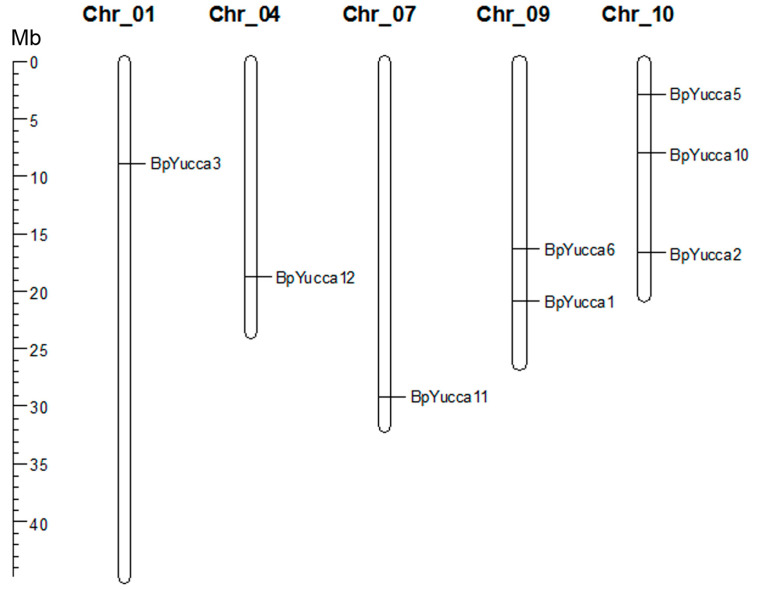
The chromosomal locations of *B. pendula Yucca* genes. The scale represents a 45-Mb chromosomal distance.

**Figure 6:**
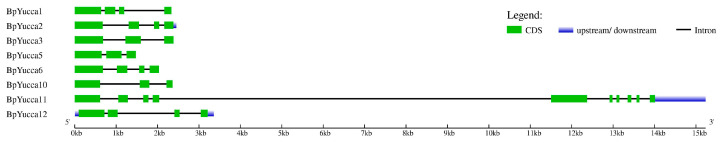
Structure of *B. pendula Yucca* genes. Intron, exon and untranslated region (UTR) are represented by black lines, green boxes and light blue boxes, respectively.

Eight genes coding for sequences homologous to Yucca proteins of *A. thaliana* and *P. trichocarpa* were identified in the silver birch genome. Putative Yucca proteins of silver birch were named according to phylogenetic analysis results (Figure 4, Table 1). *Yucca* genes of *B. pendula* were distributed on five chromosomes: Chr_01, Chr_04, Chr_07, Chr_09, Chr_10 (Figure 5). The distribution of *Yucca* genes was not uniform: Chr_10 had three *Yucca* genes (*BpYucca2*, *BpYucca5*, *BpYucca10*), Chr_09 had two genes (*BpYucca1* and *BpYucca6*), while each of Chr_01, Chr_04 and Chr_07 had only one gene. Four *Yucca* members (*BpYucca1*, *BpYucca2*, *BpYucca6*, *BpYucca12*) fad four exons, three *Yucca* genes (*BpYucca3*, *BpYucca5*, *BpYucca10*) had three exons, and *BpYucca11* had 10 exons (Figure 6).

The identified sequences contained motifs characteristic of flavin-containing monooxygenases and Yucca proteins: FMO-identifying motif “FxGxxxHxxxY/F”, two conservative “GxGxxG” motifs, (FAD-binding site and NADP-binding site), GC motif “ExxxxxAS”, ATG-containing motif 1 “WL(I/V)VATGENAE” and ATG-containing motif 2 “F/LATGY”. ATG-containing motif 1 was missing in three of the eight putative proteins. In five of the eight putative proteins, the ATG-containing motif 2 was represented by the “LATGY” sequence and in another two by the “FATGY” sequence, whereas the BpYucca12 protein was found to have two amino acid replacements within this fragment of the sequence (Table 1).

The percent identity of putative silver birch Yucca proteins shared with *A. thaliana* and *P. trichocarpa* proteins was 47.9–86.0%, excluding BpYucca11 with 27.4% and 27.2%, respectively (Table 1). Structural analysis of the Yucca11 protein showed its sequence to be longer compared to Yucca proteins of other species, and to contain the signal peptidase I, bacterial type (TIGR02227) domain, which is not typical of Yucca proteins. Previously, Alonso-Serra and others [79] demonstrated that the gene *Bpev01.c0363.g0023.m0001*, encoding BpYucca11, was expressed in silver birch trunk tissues, so we did not exclude this gene from our analysis, but the primer was chosen while keeping previous data in mind.

#### 2.2.2. GH3 Family Genes

**Figure 7:**
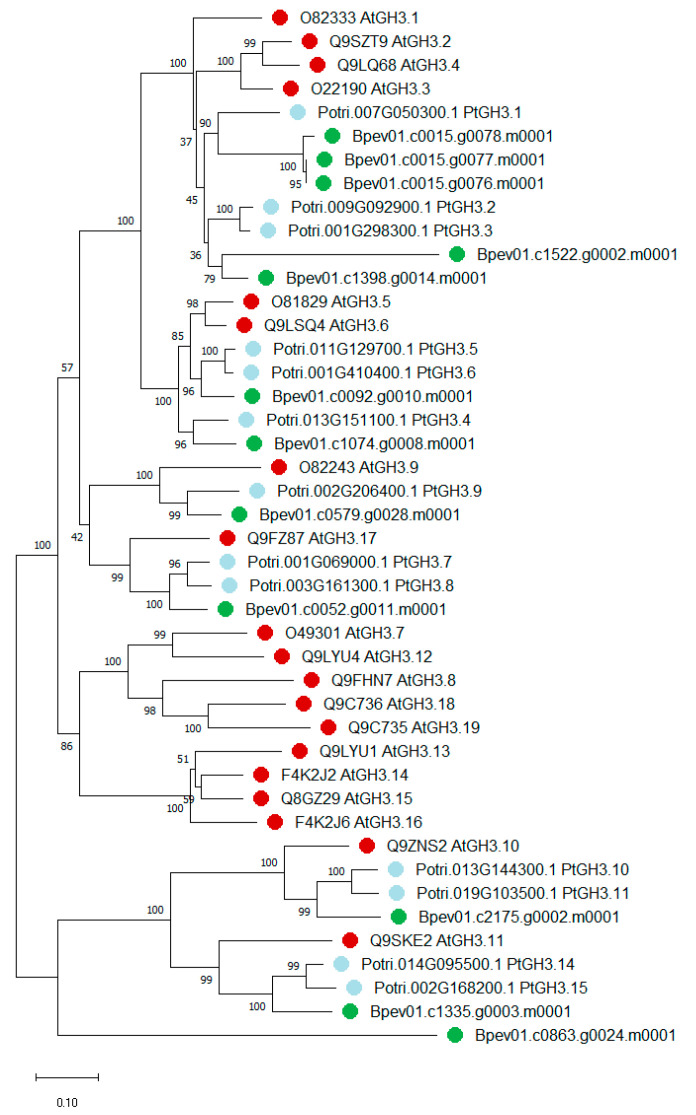
Phylogenetic relationships of GH3 proteins of *B. pendula* (green dots), *A. thaliana* (red dots) and *P. trichocarpa* (blue dots). The tree is drawn to scale, with branch lengths in the same units as those of the evolutionary distances used to infer the phylogenetic tree. Bootstrap values (1000 replicates) are shown next to the branches. The access codes of the *A. thaliana* and *P. trichocarpa* proteins in the UniProt/SwissProt and Phytozome databases are indicated next to the corresponding proteins.

Silver birch genome was found to contain 12 genes coding for sequences homologous to GH3 proteins of *A. thaliana* and *P. trichocarpa*. All the identified sequences contained domains characteristic of amido synthetases. Based on the similarity of protein amino acid sequences and substrate specificity, the family GH3 falls into three groups [80,81]. Phylogenetic analysis showed that two potential GH3 proteins in silver birch belong to group I amido synthetases, which perform the conjugation of jasmonic acid and salicylic acid. Nine birch proteins belong to the clade formed by group II amido synthetases, which are involved in auxin conjugation (Figure 7). The protein encoded by the gene *Bpev01.c0863.g0024.m0001* appears to belong to group IV amido synthetases [82]. The percent identity for protein sequences of the *Bpev01.c0863.g0024.m0001* gene product and GH3.12 protein from *Castanea mollissima*, which belongs to group IV, was 81.4%.

As this paper is dedicated to auxin homeostasis patterns, nine genes coding for group II GH3 were selected for further analysis. The characteristics of the potential proteins encoded by genes of this group are given in tabular form (Table 2). 

**Figure 8:**
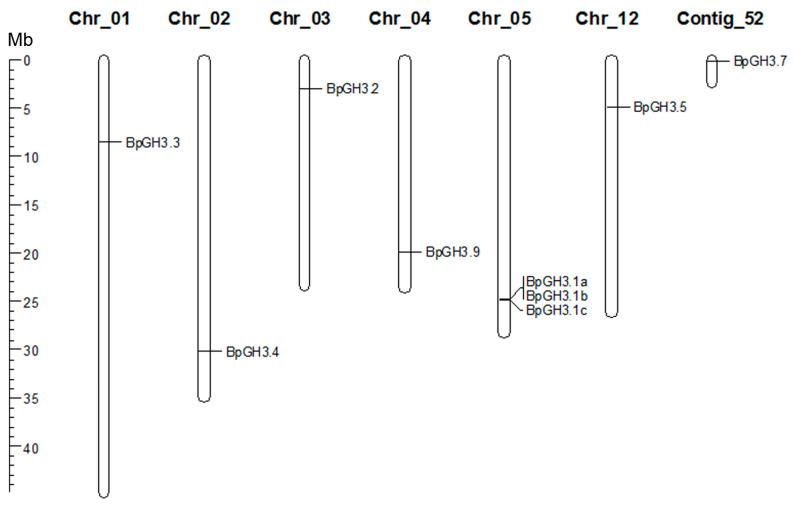
The chromosomal locations of *B. pendula* group II *GH3* genes. The scale represents a 45-Mb chromosomal distance.

**Figure 9:**
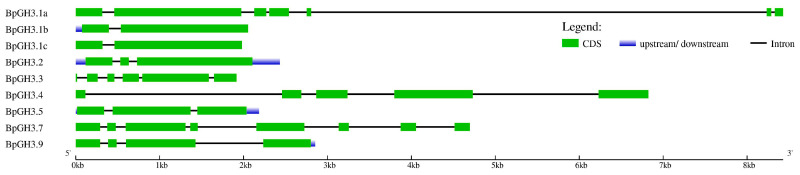
Structure of *B. pendula* group II *GH3* genes. Intron, exon and untranslated region (UTR) are represented by black lines, green boxes and light blue boxes, respectively.

The distribution of group II *GH3* genes on chromosomes was not uniform. Chr_05 had three *GH3* genes (*BpGH3.1a*, *BpGH3.1a*, *BpGH3.1a*), which were located very close to each other (Figure 8). Five group II *GH3* genes (*BpGH3.3, BpGH3.4, BpGH3.2, BpGH3.9*, and *BpGH3.5*) were distributed on chromosomes Chr_01, Chr_02, Chr_03, Chr_04 and Chr_12. The *BpGH3.7* gene was not mapped to any chromosome. The number of exons in *B.pendula* group II *GH3* genes varied from two to eight (Figure 9).

### 2.3. Expression of the Genes Involved in Auxin Biosynthesis, Transport and Conjugation

We investigated the expression of eight *Yucca* genes and nine *GH3* genes identified within this study, as well as six *PIN* genes and two *UGT* genes identified in the silver birch genome previously [69]. The analysis revealed no expression of the *Bpev01.c0015cg0065* gene coding for one of the two putative IAA–glucose synthases in any of the studied tissues. Data on the expression of all the genes selected for this study are shown in Supplementary Figure S1.

**Figure 10:**
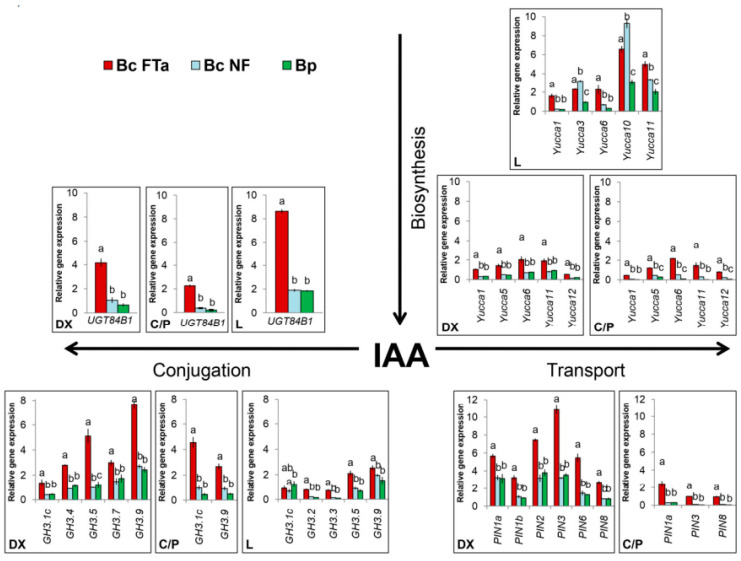
Relative gene expression of *Yucca*, *PIN*, *GH3* and *UGT* family genes that were the most active in the studied tissues. DX—differentiating xylem; C/P—layer including the cambial zone and conducting phloem; L—leaf. Bc FTa—figured parts of figured *B. pendula* var. *carelica* tree trunks; Bc NF—non-figured *B. pendula* var. *carelica* trees; Bp—*B. pendula* var. *pendula* trees.

According to the results, the expression levels of all the *Yucca* family genes, one of the two *UGT* genes and five group II *GH3* genes in leaves varied among groups of birch trees with different wood structures (Figure 10). Four group II GH3 genes (*BpGH3a*, *BpGH3b*, *BpGH3.4* and *BpGH3.7*) were not expressed in leaves of Bp trees but had low activity in leaves of Bc NF and Bc FT trees (Supplementary Figure S1). The activity of *PIN* family genes in leaves was not determined in our study.

In trunk tissues of the sampled trees, all the genes of interest were active except for one of the two *UGT* genes. A comparison of the relative expression levels of the genes in birch trees’ organs and tissues in different xylogenesis scenarios revealed that figured wood formation in Karelian birch is accompanied by a higher activity of a majority of the genes in trunk tissues, both in the differentiating xylem layer and in the layer comprising cambium and conducting phloem (Figure 10). 

**Figure 11:**
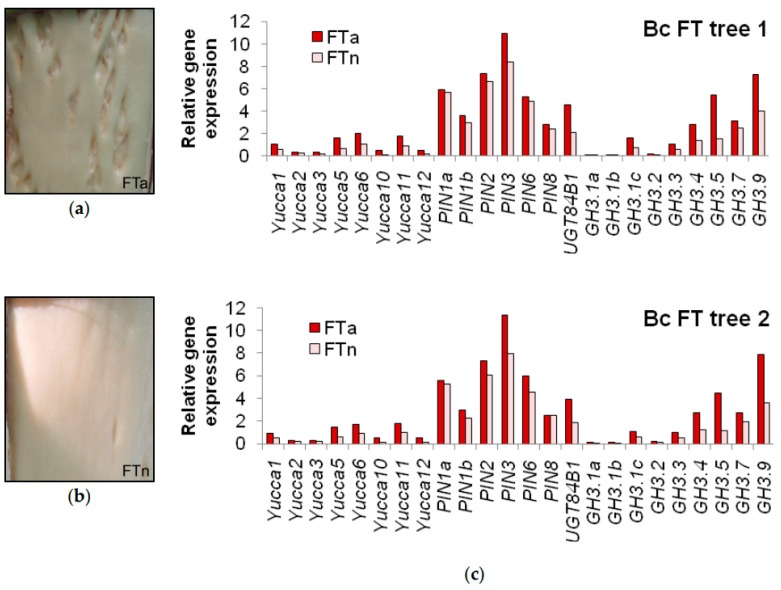
Appearance of wood in figured (**a**) and non-figured (**b**) parts of the same figured *B. pendula* var. *carelica* (Bc FT) tree trunk and relative gene expression in xylem samples obtained from two figured *B. pendula* var. *carelica* (Bc FT) trees (**c**). FTa and FTn indicate figured and non-figured parts from figured *B. pendula* var. *carelica* tree trunks.

Additionally, differentiated xylem from non-figured trunk areas (Bc FTn parts) was sampled from two Bc FT trees. Analysis revealed significant differences in the expression of some genes between figured and non-figured parts of the same trunk, suggesting that the activity of these genes is probably regulated locally (Figure 11).

### 2.4. Analysis of Promoters

Two sets of genes associated with figured wood formation in Karelian birch were selected for further analysis. Set 1 was made up of nine genes whose expression in Bc FTa parts was more than twofold higher than in Bc FTn parts of the same trunks. Set 2 was made up of 15 genes whose expression in Bc FTa parts was more than 2.5-fold higher than in the tissues of Bc NF trees (Table 3).

**Figure 12:**
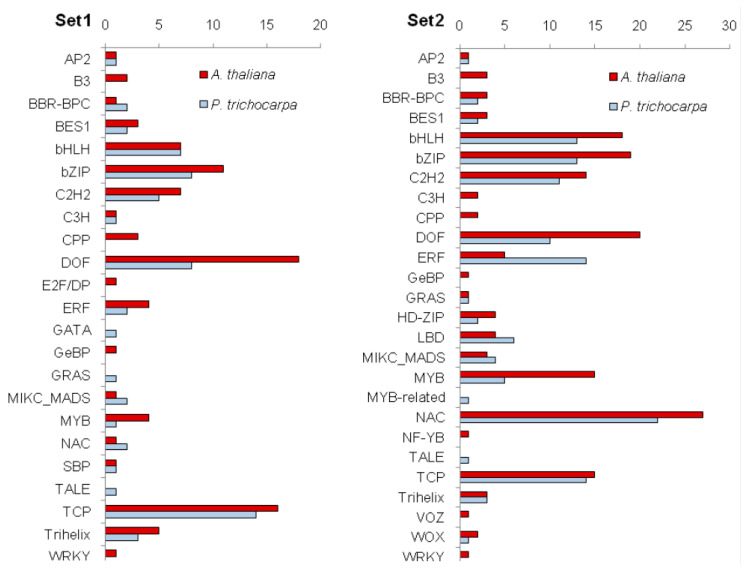
Number of overrepresented (compared to promoters in *A. thaliana* and *P. trichocarpa*) binding sites with transcription factors (TFs) from different families identified in promoters of genes from Set1 and Set2.

To gain further insights into the regulation of figured wood formation, we analysed the promoter regions of the genes included in Set 1 and Set 2 to identify transcription factor binding sites (TFBS). Analysis of the TFBS overrepresented compared to promoters in *A. thaliana* and *P. trichocarpa* revealed, respectively, 89 and 62 TFBS in gene promoters from Set 1. The greatest number of the predicted transcription factors (TFs) belonged to the families DOF, TCP and bZIP (Figure 12). For promoters of Set 2 genes, we revealed 168 and 126 TFBS were overrepresented compared to promoters in *A. thaliana* and *P. trichocarpa*, respectively. Promoters of Set 2 genes were found to have many binding sites for TFs of the families NAC, DOF and bZIP (Figure 12). The full list of predicted TFBS is provided in Supplementary Table S1.

## 3. Discussion

### 3.1. Yucca and GH3 Gene Families in the Silver Birch Genome

This paper gives a first description of the phylogenetic relationships, chromosomal localisation and structure of *Yucca* and *GH3* family genes in *B. pendula*, as well as the proteins they code for. 

Bioinformatic analysis revealed eight *Yucca* members in the genome of *B. pendula*, which is comparable to the number of *Yucca* genes in other woody plant genomes [12,13,14,17]. *Yucca* genes of birch were located on five chromosomes and contained from three to 10 exons. The structure of a majority of *B. pendula* proteins encoded by *Yucca* family genes, with the exception of BpYucca11, coincided with that of Yucca proteins in other higher plants. The sequence of the BpYucca11-encoding *Bpev01.c0363.g0023.m0001* gene should probably be analysed in more detail.

Twelve *GH3* members were identified in the genome of *B. pendula*, which is comparable with the number of genes of this family in other woody plant genomes [40,41,43,82]. Of the twelve *GH3* family genes, two were classified as belonging to group I *GH3*s, which performs the conjugation of jasmonic acid and salicylic acid, while nine were group II *GH3*s genes, which are involved in auxin conjugation [80,81]. One gene appears to belong to group IV *GH3*s, recently identified in woody plants, whose function is yet unknown [82]. As this paper is dedicated to auxin homeostasis patterns, nine genes coding for group II GH3 were selected for further analysis. Group II *GH3* genes in birch were located on six chromosomes and one contig, and contained between two and eight exons. Although three genes encoding proteins homologous to *A. thaliana* and *P. trichocarpa* GH3.1 proteins were closely localised on the same chromosome, analysis showed that the structure of these genes varied significantly. 

The results for the *Yucca* and *GH3* members identified in the genome of *B. pendula* provide the basis for further studies of auxin-related genes in non-model woody plants.

### 3.2. Conducting Tissue Structure in Karelian Birch Trunk Indicates a Reduced Free Auxin Level

The process of xylem vessel differentiation is believed to be the most sensitive to the level of free (physiologically active) auxin [23,26,83]. Numerous experiments with woody plant shoots and transgenic plants have demonstrated that the density of vessels in the xylem decreases where the auxin concentration is low [84,85,86,87,88,89,90]. Moreover, there is some evidence indicating that the differentiation of phloem sieve elements is induced by auxin, albeit in lower concentrations when compared with xylem vessel differentiation [5,26]. 

It has previously been demonstrated that, after many years of figured wood formation in adult Karelian birch trees (25–30-year-old), the vessel density in the anomaly areas is much lower than in the wood of common silver birch, while, in non-figured parts, vessel density is slightly lower [58,59,69]. Analysis of auxin content revealed a reduction in the concentration of free auxin and a rise in the concentration of auxin conjugates in the areas of anomalies [64], thus supporting the evidence that the development of anomalous conducting tissues in Karelian birch trunks is closely associated with auxin inactivation.

For the current study younger, 13–14-year-old common silver birch and Karelian birch trees were selected. One of the earliest signs of wood figure expression, as revealed by microscopic analysis, is the disruption of the formation of highly specialised conducting elements in the xylem and phloem—vessels and sieve tubes. Thus, conducting tissues in anomaly areas in Karelian birch trunks sampled for molecular genetic analysis show traits of an auxin-deficient phenotype.

### 3.3. Expression of the Main Genes Involved in Auxin Biosynthesis, Transport and Conjugation during the Formation of Wood Typical of Birch Species

The expression level of *Yucca*, *PIN*, *UGT* and *GH3* genes varied among organs and tissues of the trees forming straight-grained wood typical of birch (Bp trees and Bc NF trees). A majority of *Yucca* genes were more active in leaves compared to trunk tissues, which is in good agreement with data from the literature arguing that the main localisation of auxin biosynthesis is leaves and shoot apexes [1,9,26], as well as with data on *Yucca* expression in the organs of other woody plants [12,17]. Among genes of this family, the expression level of *BpYucca5*, *BpYucca6* and *BpYucca11* was the highest in trunk tissues, potentially suggesting that these genes may be involved in the generation of local auxin gradients in the cambial zone [18,53].

The expression level of *PIN* family genes in trees forming normally structured wood was higher on the differentiating xylem side compared to the tissue layer comprising the cambial zone and the conducting phloem. Some studies have demonstrated that polar auxin transport is mainly channelled via cambial zone cells and the nearby differentiating derivatives [91,92], which agrees with the expression of the genes encoding “long” PIN proteins [32,93,94]. Sampling in our study was done during active xylem differentiation, while the conducting phloem in the birch trunk would usually be already fully formed by this time [95], which is probably the reason for the higher activity of *PIN*s on the differentiating xylem side. The highest expression level was demonstrated by three genes coding for “long” PIN proteins—*BpPIN1a*, *BpPIN2* and *BpPIN3*.

Interestingly, the available data indicate that, in normally growing and developing woody plants, the activity of the genes coding for the main auxin conjugation enzymes differs in leaves and in trunk tissues. Thus, auxin inactivation in leaves primarily results in the formation of IAA–glucose conjugate, whereas higher activity in trunk tissues is demonstrated by *GH3* genes encoding IAA–amino synthases. To the best of our knowledge, there has been no comparative study of the expression profiles of the genes involved in auxin conjugation with sugar and amino acids in different organs of adult woody plants before. It has been shown for rice, however, that the *OsIAGT1* gene encoding IAA–glucose synthase is the most active in mature leaves compared to other plant parts, whereas the expression of some *GH3* family genes can differ significantly between leaves and the shoot [48,96]. We suppose these differences in the activity of the genes encoding auxin conjugation enzymes to be associated with the different functions of ester and amide conjugates.

### 3.4. Specific Traits of Expression of the Genes in Question during Figured Wood Formation in Karelian Birch

It is in early development stages that Karelian birch saplings feature a more active carbohydrate metabolism compared to common silver birch [97,98]. It has been demonstrated for trees of different ages that figured wood formation in Karelian birch is associated with a rise in the amount of photoassimilates transported via the phloem and with high hexose influx to cells in the differentiating xylem [58,63,67,69]. It can thus be concluded that an altered metabolic status of differentiating trunk tissues is strong biochemical marker of abnormal xylogenesis. In view of the above, we believe the elevated activity of the genes involved in auxin biosynthesis, transport and conjugation in Bc FT trees may be due to the effect of sugars on gene expression. Soluble sugars are known to be able to transcriptionally regulate a significant number of auxin-related genes. In both herbaceous and woody plants, a rise in sugar (glucose, sucrose) level caused an upregulation of *Yucca*, *PIN* and *GH3* genes [99,100,101,102,103,104,105]. 

Overexpression of *Yucca* genes in different species resulted in the elevation of IAA content and the production of auxin-overproduced phenotypes [17,18,106,107]. Although the background for figured wood formation is the elevated activity of auxin-biosynthesis genes, the structure of conducting tissues in figured-wood parts still demonstrated signs of an auxin-deficient phenotype. The expression of auxin-responsive genes (*PIN*, *GH3*) also being elevated in figured parts of Karelian birch trunks probably cannot be explained by a loss of auxin sensitivity by the tissues and impaired auxin signalling, although this assumption also needs to be tested experimentally.

In turn, overexpression of various *GH3* genes was demonstrated to entail a reduction in free (physiologically active) auxin levels and the generation of auxin-deficient phenotypes [39], and to hinder the formation of the hormone’s gradient in the cambial zone [42]. A negative correlation between the expression of some *GH3* genes and foliar IAA content was found in the closely related species *B. platyphylla* [43]. Transgenic *A. thaliana* and *Oryza sativa* plants overexpressing the IAA–glucose synthase gene simultaneously exhibited a reduction in free auxin level and morphological changes characteristic of auxin-deficient phenotypes [48,108,109]. Thus, overexpression of auxin conjugation coding genes *GH3* and *UGT84B1* appears to have a greater effect on figured wood formation in birch than overexpression of *Yucca* genes. The likely reason for that is differences in the kinetics of the enzymatic reactions of auxin biosynthesis and metabolisation. There is some evidence that Yucca flavin-dependent monooxygenases, which catalyse the rate-limiting step in IAA synthesis, have slow kinetics [110,111], whereas IAA–amino synthases encoded by genes of the family *GH3* are described as enzymes with fast kinetics [80,112,113,114]. 

Auxin can be inactivated not only through its biochemical transformations, but also by localising it in the cell so that it can no longer perform its regulatory functions. Of much interest in this connection is the study of the role of so-called “short” PIN proteins in auxin inactivation and figured wood formation in Karelian birch. The *BpPIN6* and *BpPIN8* genes, classified previously as encoding “short” PIN proteins [69], were upregulated in the differentiating xylem of figured Karelian birch trees. “Short” PIN proteins are believed to be involved in auxin transport between the cytoplasm and ER; this process may have a role in maintaining the free auxin concentration in the cytoplasm [115,116,117]. It has been demonstrated for *Arabidopsis* and tobacco plants that *PIN8* overexpression promotes the accumulation of free auxin and IAA–glucose conjugate in cells, at the same time preventing auxin-dependent activation of gene expression, presumably because the hormone is primarily accumulated in ER [118]. Overexpression of the gene encoding the “short” PIN5 protein led to a marked decline of free auxin in tissues and to accumulation of the amide conjugates IAA–Asp and IAA–Glu, as well as reduced auxin transport out of cells [119].

Figured wood formation was also accompanied by overexpression of the genes encoding “long” PIN proteins involved in intercellular auxin transport, pointing to activated auxin transport to other organs and tissues. The most significantly upregulated was the *BpPIN3* gene, which is in agreement with previously obtained data on older Karelian birch trees [69]. *PIN3* expression in woody plants’ shoots and trunk tissues is mainly observed in parenchyma cells in the bark periphery, as well as in xylem radial parenchyma [35,79,93,120], suggesting the PIN3 protein may be involved in auxin radial intercellular transport [92,121]. It has been hypothesised that overexpression of *BpPIN3* may promote the emergence of various auxin transport directions through tissues in figured-wood parts of Karelian birch trunks [69]. However, it was shown that “long” PINs affected auxin distribution in tissues not only by modulating the expression of corresponding genes but also through the alteration of their localisation on the membrane [29,99,120]. Therefore, the study of changes in the localisation of PIN proteins during the formation of figured wood is of great interest for future research.

**Figure 13:**
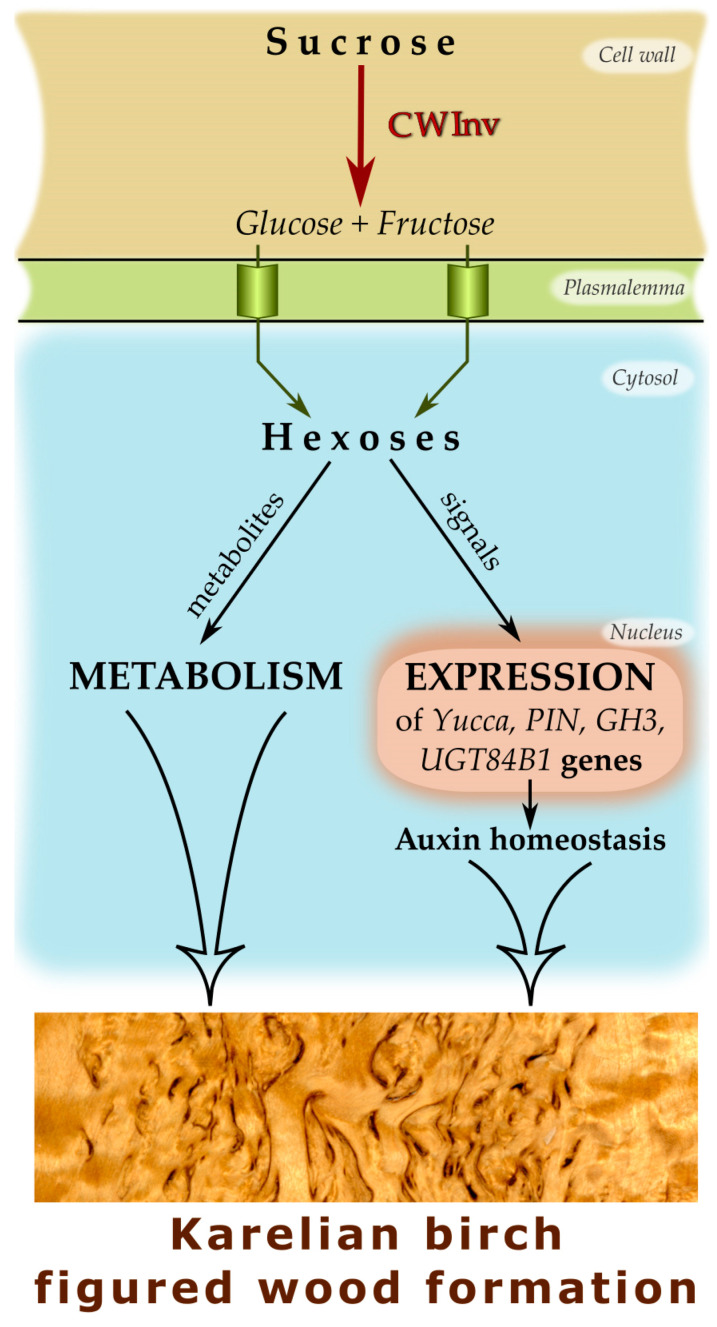
Flowchart of possible interaction between sugars and auxin during figured wood formation in Karelian birch trunks. CWInv—cell wall invertase.

Based on the results obtained through this study and previously [63,64,65,66,67,68,69], the possible mechanism of hexose influence on the expression of auxin-related genes and figured wood formation in the trunks of Karelian birch trees has been summarised in a flowchart (Figure 13).

Differences in the expression of the investigated genes between Bc NF trees and Bc FT trees support the statement that figured wood formation in Karelian birch trees is tightly connected with sugar distribution between different tree organs, namely crown and trunk [63,98,122]. Bc NF trees had a higher expression of *BpYucca3*, *BpYucca10* and *BpYucca12* in leaves compared to Bp trees. Since *PIN*, *UGT84B1* and *GH3* expression in trunk tissues of Bc NF trees was much lower than in Bc FT trees and the same as in Bp trees, it is probable that sugar in this group of trees is mostly retained in the crown, and auxin biosynthesis is thus activated. The higher level of sugar and auxin in the crown of Bc NF trees must be the reason for the previously detected more active growth of leaves and shoots in this group of trees as opposed to Bp trees [123]. The active acceptors of assimilates in Bc FT trees are the differentiating trunk tissues [98,122], and the richest wood figure is formed at confluences of phloem flows, where photoassimilate concentrations are locally elevated [63,67]. In our study, a majority of *Yucca* and *GH3* genes were overexpressed only in Bc FTa parts (i.e., in areas with elevated sugar content) but not in Bc FTn parts, suggesting that their expression is regulated locally rather than at the whole-plant level.

### 3.5. Identification of Transcription Factors Potentially Involved in Gene Expression Regulation during Figured Wood Formation in Karelian Birch

As it is known that the influence of sugars on gene expression could be mediated through the different sugar-responsive transcription factors [123,124,125,126], we conducted an analysis of overrepresented TFBS in promoters of genes whose expression was significantly upregulated during figured wood formation. Quite a few binding sites for TFs of the families NAC, DOF, TCP and bZIP, as well as some others, were identified. TFs belonging to the families NAC and DOF are important players in conducting tissue formation [127,128,129]. Some NAC and DOF TFs regulate the differentiation of specific types of xylem cells for fibres and vessels [130,131,132,133]. Binding sites for several fibre- and vessel-specific TFs were detected in promoters of genes belonging to Set1 and Set2. Moreover, in the DOF family there is a small group of mobile transcription factors—PEAR proteins—which are involved in radial growth regulation [134]. Binding sites of four out of six PEAR proteins (DOF3.2, DOF5.1, DOF5.6/HCA2, DOF2.4) were detected in promoters of genes from Set1 and Set2. Previously, binding sites of three PEAR proteins (DOF3.2, DOF5.1, DOF5.6/HCA2) were identified in promoters of the genes *CWInv1*-*CWInv3* coding for cell wall invertase in silver birch, whose activity was also significantly elevated during figured wood formation in Karelian birch [135]. TCP TFs participate in regulating plant organs’ morphogenesis and translate various endogenous and exogenous signals into growth responses [136,137]. bZIP are a large group of TFs with functions in the differentiation of various organs and tissues and in the plant response to biotic and abiotic environmental factors [138].

Some TFs in the above families are involved in auxin signalling. It was demonstrated for *P. tremula* × *tremuloides* plants, for instance, that auxin downregulates NAC TFs associated with fibre differentiation and induced vessel-specific NACs [139]. Mutant *A. thaliana* Dof5.1-D plants overexpressing *PEAR2/DOF5.1* have a reduced level of auxin biosynthesis and signalling compared to wild-type plants [140]. TCPs are known to modulate auxin synthesis, transport and response [141]. bZIP binding sites in promoters of auxin-responsive genes can participate in the fine tuning of their auxin-induced transcription [142,143,144]. Furthermore, DOF TFs and bZIP TFs participate in sugar signalling [126,145], so it would be interesting to study the potential involvement of these two families in regulating alternative xylogenesis scenarios. Nevertheless, notice should be given to the fact that the transcription factors identified in this work have multiple downstream targets and could regulate other hormone pathways as well.

## 4. Materials and Methods 

### 4.1. Choice of Trees and Sampling

The trees selected for the study were common silver birch and Karelian birch specimens raised from seeds collected from controlled pollinated plus trees (Forelia OY, Häkkilä, Finland). The sample trees were 13–14 years old. They were all growing in the same soil and climatic conditions in one test plot in the KarRC RAS Agrobiological Station situated 2 km away from Petrozavodsk.

Trunk tissue and leaves were collected during the period of high cambial activity, early in July 2019, from three specimens of (1) common silver birch trees (*B. pendula* var. *pendula*), (2) Karelian birch trees with straight-grained non-figured wood (non-figured *B. pendula* var. *carelica* trees), and (3) Karelian birch trees with richly figured wood (figured *B. pendula* var. *carelica* trees). All the Karelian birch trees were high stemmed, without signs of stunted growth. In figured Karelian birch trees, tissues were collected from (a) trunk areas with figured wood (Bc FTa parts), and (b) trunk areas with non-figured wood (Bc FTn parts). 

For trunk tissue sampling, rectangular bark pieces were cut out on the trunk with a sharp knife 130 cm above the ground, and carefully separated from wood. A thin layer of xylem tissue comprising the zone of cell expansion and differentiation was scraped off the exposed wood surface with a razor. A layer of tissues comprising the conducting phloem and the cambial zone was scraped off the inner surface of the bark. Samples were collected under the microscope control. The tissues were frozen in liquid nitrogen directly after sampling. Bark and wood samples for microscopic analysis were taken from the immediate vicinity of the previous samples. In this case, cubic blocks with 1 cm sides, containing 2–3 annual increments, were chiselled out of the trunk.

### 4.2. Microscopy

Sample preparation for microscopy was done as described previously [69]. Microscopic analysis was carried out under an AxioImager A1 light microscope (Karl Zeiss, Oberkochen, Germany) equipped with an ADF PRO03 camera. Images were processed with ADF Image Capture software (ADF Optics, Wuhan, China). Anatomical measurements were made following available guidelines [146,147,148]. 

### 4.3. Total RNA Isolation and Complementary DNA Synthesis

To isolate total RNA, a 50–100 mg aliquot of plant tissue was ground in liquid nitrogen. The isolation procedure was run using two lysis buffers (based on cetyl trimethylammonium bromide (CTAB) and sodium dodecylsulphate (SDS)) and chloroform-isoamyl alcohol mixture (24:1) according to the technique described in [149]. Tissue lysate was additionally treated with RNase inhibitor to prevent sample degradation and with DNase (Syntol, Moscow, Russia) to remove genomic DNA impurities. The tissue/buffer ratio was 1:10. The CTAB extraction buffer contained 100 mM Tris–HCl (pH 8.0); 25 mM EDTA, 2 M NaCl, 2% CTAB, 2% PVP, and 2% mercaptoethanol (added immediately before use); the SDS extraction buffer contained 10 mM Tris–HCl, 1 mM EDTA, 1 M NaCl, and 0.5% SDS.

RNA was precipitated by 100% isopropanol. RNA precipitate was dissolved in an adequate amount of RNase-free water. The integrity of the resultant RNA was tested by electrophoresis in 1% agarose gel. RNA concentration was measured spectrophotometrically (microplate reader SPECTROstar NANO, BMG Labtech, Ortenberg, Germany) through absorbance at 260 nm wavelength. The purity of the specimen was evaluated by the optical density ratio measured at 230, 260 and 280 nm (A260/A280 and A260/A230). The absence of genomic DNA impurities was verified by PCR with the RNA sample as the matrix.

cDNA was synthesised following manufacturer’s protocol using the reverse transcription reagent kit MMLV RT (Evrogen, Moscow, Russia) in 20 µL of reaction mixture containing 6 µL RNA matrix (1 µg), 1 µL Oligo(dT) primer and 1 µL random primer (Randoom (dN)10-primer) (20 µM), 1 µL reverse transcriptase (MMLV revertase) (100 units), 4 µL of 5x buffer for the first chain synthesis cDNA (280 mM Tris-HCl, pH 8.7; 37 5 mM KCl; 30 mM MgCl_2_), 2 µL dNTP mixture (10 mM of each), 2 µL DDT (20 mM), 3 µL sterile RNase-free water. The RT reaction was performed in a QuantStudio 5 amplifier (Thermo Scientific, Waltham, MA, USA).

### 4.4. Gene Search in the Silver Birch Genome

Previously, six *PIN* family genes and two genes coding for proteins homologous to IAA–glucose synthase in *Arabidopsis* have been identified in the silver birch genome [69].

The search for *Yucca* and *GH3* genes was carried out using the published genome of *Betula pendula* Roth [150]. To this end, the CDS of *Arabidopsis thaliana* and *Populus trichocarpa Yucca* and *GH3* genes and the amino acid sequences of corresponding proteins were obtained from The Arabidopsis Information Resource (TAIR) database (release 13, https://www.arabidopsis.org) and Phytozome database (http://www.phytozome.net/poplar, release v3.0), respectively. The resulting sequences were then used as a BLAST search query across the genome of *B. pendula* var. *pendula* (release 1.2, https://genomevolution.org/coge) to identify homologous sequences.

The structures of candidate proteins were predicted using the National Centre for Biotechnology Information (NCBI) resource (http://www.ncbi.nlm.nih.gov/Structure/cdd/cdd.shtml) [151]. Phylogenetic analysis was carried out using MEGA X software [152]. Multiple sequence alignment for the protein sequences was performed using ClustalW. Phylogenetic trees were constructed using the neighbour-joining method based on the Poisson correction model with 1000 bootstrap replicates [153,154,155]. The percent identity of *B. pendula* and *A. thaliana* proteins was determined using the EMBOSS Needle online tool (https://www.ebi.ac.uk/Tools/psa/emboss_needle/).

The chromosome locations of Yucca and GH3 family members in B. pendula (2*n* = 28) were investigated using MapChart software [156]. The gene structures were analysed using the Gene Structure Display Server (http://gsds.cbi.pku.edu.cn/) [157].

### 4.5. Determination of Gene Expression Levels (Real-Time qPCR)

Specific primers (Evrogen, Russia) for amplifying the target and reference genes were constructed using BeaconDesigner 8.21 software (PREMIER Biosoft) (Table 4). The reference genes for calculating the relative expression of the target genes were *GAPDH* and *EF1a* [158]. To make sure that the reaction yielded one product, the melting curve was analysed and electrophoresis of quantitative PCR products in 8% acrylamide gel with ethidium bromide staining was performed.

Samples were amplified in QuantStudio 5 amplifier (Thermo scientific, USA) using kits with SYBR Green (qPCRmix-HS SYBR) intercalating dye (Evrogen, Russia). Real-time PCR was run in 25 µL of reaction mixture containing 5 µL qPCRmix-HS SYBR, 1 µL of each of forward and reverse primers (0.4 µM) (Syntol, Russia), 2 µL cDNA matrix, and 16 µL of deionised nuclease-free water. The final cDNA content in the reaction medium for all samples was 100 ng, as recommended in manufacturer’s protocol. The PCR protocol was 95 °C for 5 min, then forty 15 s cycles at 95 °C for denaturation, 30 s at 55 °C for annealing, and 30 s at 72 °C for elongation. Negative control was applied for each pair of primers, i.e., PCR was run without cDNA matrix.

PCR product specificity was evaluated by analysing melting curves. To determine the efficiency (E), PCR was performed with each pair of primers after serial 10-fold cDNA dilutions (10^−1^, 10^−2^, 10^−3^, 10^−4^ and 10^−5^). Cycle threshold (Ct) values were determined by plotting calibration curves in Microsoft Office Excel based on correlation coefficients R and slope (k), which can be calculated from the calibration curves of Ct values for each gene. The efficiency of PCR amplification (E) was calculated by the formula

E = (10^(^−1^/k) − 1) × 100. (1)

The expression of specific genes was represented in relative units (r.u.) normalised to the expression of the reference gene. Based on the expression levels of two reference genes (*GAPDH* and *EF1a*) the arithmetic mean Ct of the reference genes was calculated for each sample. 

Relative quantification (RQ) of gene transcription followed the formula

RQ = E^−ΔCt^, (2)

where E is PCR efficiency,

ΔCt = Ct (target gene) − Ct (arithmetic mean of the two reference genes).(3)

The data produced by PCR were analysed using the Relative Expression Software Tool 2009 V.2.0.13 (REST 2009).

All assays were performed at the Core Facility of the Karelian Research Centre RAS.

### 4.6. Analysis of Promoters

The 2-Kbp upstream promoter sequences from the start codon (ATG) of all the studied genes were used for the analysis of cis-acting elements. To identify over-represented TFBS, the frequency of cis-acting elements occurrence was analysed and statistically estimated based on the occurrence of cis-acting elements in promoters of the model organisms *A. thaliana* and *P. trichocarpa* using the Regulation Prediction tool from PlantRegMap (http://plantregmap.cbi.pku.edu.cn/) [159].

## 5. Conclusions

We have identified the key genes involved in auxin biosynthesis, transport and conjugation in the *Betula pendula* Roth genome and investigated their expression in trunk tissues and leaves of adult birch trees with different anatomical structure of the wood (figured and non-figured trees of Karelian birch and common silver birch trees). 

Figured wood formation in Karelian birch was accompanied by overexpression of the genes involved in auxin homeostasis, suggesting that this hormone is an important actor in the formation of conducting trunk tissues with abnormal structures in woody plants. In spite of the higher expression of the genes encoding auxin biosynthesis enzymes, conducting tissues in trunk parts with figured wood exhibited traits of an auxin-deficient phenotype, which agrees with evidence on reduced free auxin content in anomaly areas in Karelian birch trunk. The probable cause of the decline in physiologically active auxin is its inactivation through conjugation with sugars and amino acids, as well as deposition in intracellular compartments.

We identified many binding sites with various TFs in promoters of the genes whose expression was significantly upregulated during figured wood formation. Most of them belong to the NAC, DOF, TCP, and bZIP families.

Undoubtedly, the formation of figured wood in Karelian birch is determined by the combination of multiple processes. Nevertheless, the data obtained are consistent with the hypothesis suggested before that anomalous figured wood formation in Karelian birch may be associated with the sugar induction of auxin conjugation.

## Figures and Tables

**Table 1:** Characteristics of Yucca members in birch.

Gene ID	*B. pendula* Protein Name	Protein Length (aa)	Motif Sequences		Closest Homolog in *A. thaliana* (% Identity)	Closest Homolog in *P. trichocarpa* (% Identity)
			ATG-Containing Motif 1	ATG-Containing Motif 2		
*Bpev01.c0000.g0045.m0001*	BpYucca5	414	WLVVATGENAE	LATGY	AtYucca9 (74.3%)	PtYucca8 (86%)
*Bpev01.c0039.g0010.m0001*	BpYucca10	330	FLIAATGENSE	FATGY	AtYucca10 (49.1%)	PtYucca9 (47.9%)
*Bpev01.c0118.g0037.m0001*	BpYucca1	391	WFIVATGENAE	LATGY	AtYucca4 (65.9%)	PtYucca1 (71.2%)
*Bpev01.c0171.g0019.m0001*	BpYucca12	381	FLVVATGEATD	FCTGF	AtYucca10 (48.1%)	PtYucca12 (68.5%)
*Bpev01.c0363.g0023.m0001*	BpYucca11	802	FLVVATGENSE	FATGY	AtYucca10 (27.4%)	PtYucca12 (27.2%)
*Bpev01.c0389.g0021.m0001*	BpYucca2	421	WLIVATGENAE	LATGY	AtYucca2 (67.9%)	PtYucca6 (70%)
*Bpev01.c0547.g0012.m0001*	BpYucca3	426	WLVVATGENAE	LATGY	AtYucca7 (71.4%)	PtYucca3 (79.1%)
*Bpev01.c1498.g0001.m0001*	BpYucca6	424	WLVVATGENAE	LATGY	AtYucca6 (68.2%)	PtYucca2 (73.5%)

**Table 2:** Characteristics of GH3 members in birch.

Gene ID	*B. pendula* Protein Name	Protein Length (aa)	Closest Homolog in *A. thaliana* (% identity)	Closest Homolog in *P. trichocarpa* (% identity)
*Bpev01.c0015.g0076.m0001*	BpGH3.1a	806	AtGH3.1 (55.2%)	PtGH3.1 (59.9%)
*Bpev01.c0015.g0077.m0001*	BpGH3.1b	612	AtGH3.1 (73.0%)	PtGH3.1 (79.1%)
*Bpev01.c0015.g0078.m0001*	BpGH3.1c	612	AtGH3.1 (72.0%)	PtGH3.1 (78.1%)
*Bpev01.c0052.g0011.m0001*	BpGH3.7	753	AtGH3.17 (57.4%)	PtGH3.7 (66.2%)
*Bpev01.c0092.g0010.m0001*	BpGH3.5	612	AtGH3.6 (84.3%)	PtGH3.5 (87.7%)
*Bpev01.c0579.g0028.m0001*	BpGH3.9	595	AtGH3.9 (72.1%)	PtGH3.9 (81.5%)
*Bpev01.c1074.g0008.m0001*	BpGH3.4	749	AtGH3.6 (64.2%)	PtGH3.4 (67.5%)
*Bpev01.c1398.g0014.m0001*	BpGH3.2	598	AtGH3.1 (81.5%)	PtGH3.3 (86.8%)
*Bpev01.c1522.g0002.m0001*	BpGH3.3	495	AtGH3.1 (50.0%)	PtGH3.3 (53.9%)

**Table 3:** List of genes included in Set 1 and Set 2.

Process	Gene family	Set 1	Set 2
IAA biosynthesis	*Yucca*	*BpYucca5*	*BpYucca1*
		*BpYucca10*	*BpYucca5*
		*BpYucca11*	*BpYucca6*
		*BpYucca12*	*BpYucca10*
			*BpYucca12*
IAA transport	*PIN*		*BpPIN1b*
			*BpPIN3*
			*BpPIN6*
			*BpPIN8*
IAA conjugation	*GH3*	*GH3.1a*	*GH3.1c*
		*GH3.1b*	*GH3.2*
		*GH3.4*	*GH3.4*
		*GH3.5*	*GH3.5*
			*GH3.9*
	*UGT*	*UGT84B1*	*UGT84B1*

**Table 4:** List of primers for the RT-PCR reaction.

Gene Name	Forward Primer	Reverse Primer
*BpYucca1*	ATTCAATAATCTTAGCAACTG	TGTATAAGTCCGTTCTCT
*BpYucca2*	GTTAGAGATTCGGTTCAT	TGACATTAGAAGCAAGAA
*BpYucca3*	TATTAGTTGTTGGTTGTG	ATATCAGCAATATCTTATCG
*BpYucca5*	GGAGTACAAGAACACCAT	GCAAGAACAACTGAATCAA
*BpYucca6*	TATACTCAGTTGGGTTTA	AAATGAAATGAAGAATAAGG
*BpYucca10*	AGGTGGAAGTATATGTTG	TGTAATGGTTGGAATGTA
*BpYucca11*	TAGAAGGAAGTATGGAAG	GTTGGAATGGAGAATATC
*BpYucca12*	GCTTCAAGAGGTCCACAA	TAGTCCAACGCAGTATAATCC
*BpPIN1a*	AAGGCTTATTCTCATCAT	GTATGGAAATGGACTTTG
*BpPIN1b*	CTCTCCTTTCCTTTCACTTC	ACCACCAAGACGATTACT
*BpPIN2*	AATAGAGGAAGGATTGAAG	GACTTGAGTAGGTGTTAG
*BpPIN3*	GTTCTCGTCCACTATTACTAA	GCAACTCCTTAGCATCAT
*BpPIN6*	TCATATCTCAGAACAATCC	AAGAGTGATAAGCCAATC
*BpPIN8*	CATTATATGTTGTGATGATAGTAG	AGCAGAGGAATTGAGTAT
*BpGH3.1a*	GAAGCAATGGATATGATT	CAATTAGTGTCTAGGATG
*BpGH3.1b*	AGTGTTCAGAAGAGAATT	GAGTGTTGGTATGTATTG
*BpGH3.1c*	ATTCCTACAACTCAACTG	ATTCGTGTGATTATACCTT
*BpGH3.2*	TTTATTGCCAAAGAATGC	AGATTGACTCCGAAATAG
*BpGH3.3*	CAGACTCTGGATCACTAT	CATTATGGTGTACGAGAC
*BpGH3.4*	TTATAGTAACAGGAACAATG	GCAAGAACTCATAATAACA
*BpGH3.5*	AGTATGTGGATGTTATTGT	AAGAGGATTAAGGTTGAC
*BpGH3.7*	CTGTTGTGAATTATGAAGA	AAGGATGTTGTAGAAGAA
*BpGH3.9*	TATCTCAACAACTATCTCAA	TCACCATTAGCAATTCTT

## Supplementary Materials

The following are available online at https://www.mdpi.com/2223-7747/9/11/1406/s1, Figure S1: Relative gene expression of all *Yucca*, *PIN*, *GH3*, and *UGT* family genes, Table S1: List of enriched TFs in the input gene lists of Set 1 and Set 2.
