# Supplementary material for: Expression Analysis of Key Auxin Biosynthesis, Transport, and Metabolism Genes of Betula pendula with Special Emphasis on Figured Wood Formation in Karelian Birch
Source: Plants (Basel). 2020 Oct 22;9(11):1406. doi: 10.3390/plants9111406 (PMC7690449; doi:10.3390/plants9111406)
Supplement: Supplementary file 1 [file plants-09-01406-s001.zip › Supplementary Figure S1.pdf]

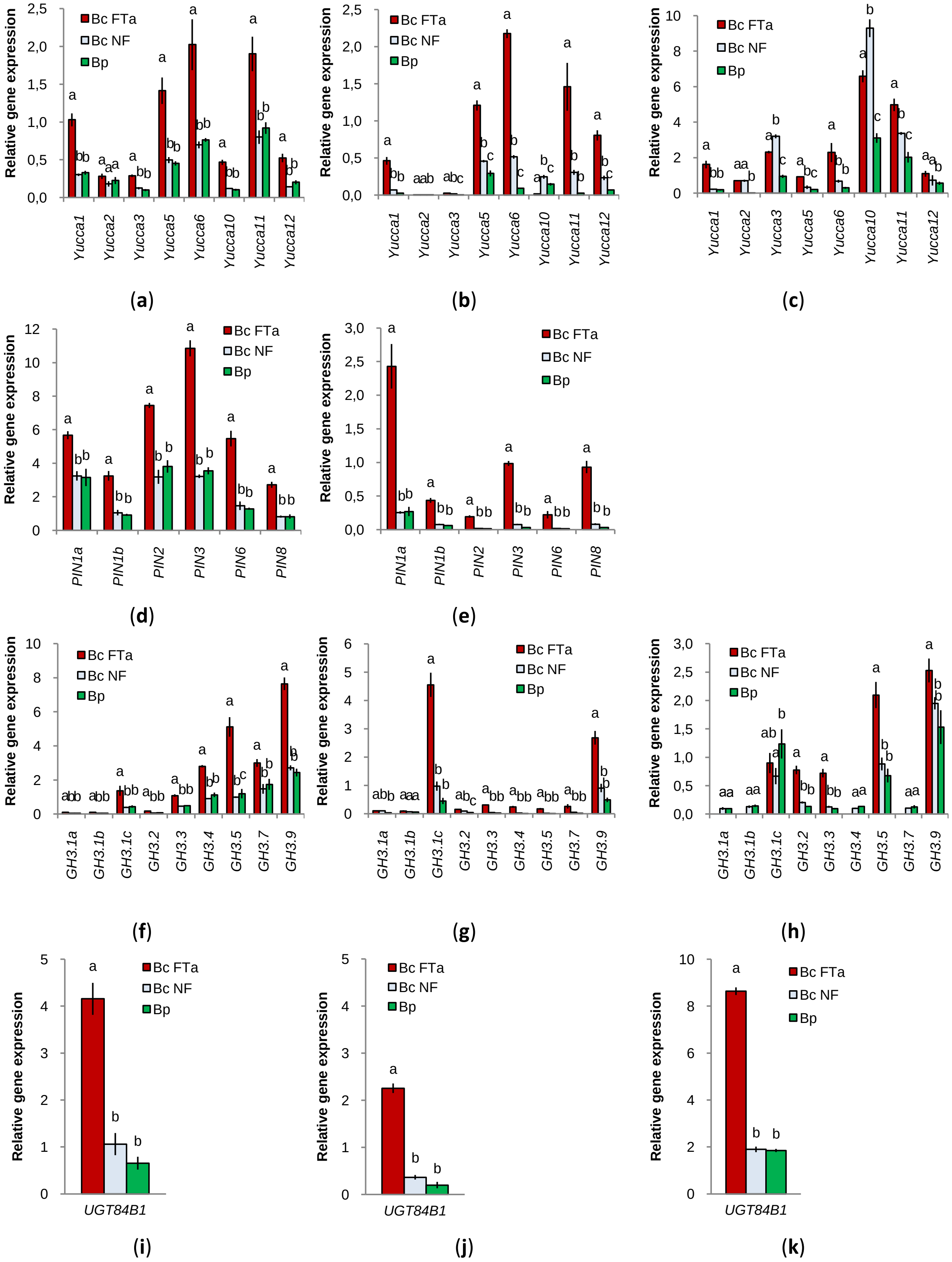

Supplementary Figure S1. Relative gene expression of all *Yucca*, *PIN*, *GH3* and *UGT* family genes: (**a**), (**d**), (**f**), (**i**) differentiating xylem; (**b**), (**e**), (**g**), (**j**) tissues layer included cambial zone and conducting phloem; (**c**), (**h**), (**k**) leaf. Bc FTa – figured parts of figured *B. pendula* var. *carelica* tree trunks; Bc NF - non-figured *B. pendula* var. *carelica* trees; Bp - *B. pendula* var. *pendula* trees.
